# Relevance of Haemodynamics in Treating Pre-eclampsia

**DOI:** 10.1007/s11906-017-0766-6

**Published:** 2017-08-23

**Authors:** Christoph Lees, Enrico Ferrazzi

**Affiliations:** 1 Imperial College London, London, UK; 2 Centre for Fetal Care, Queen Charlotte’s and Chelsea Hospital, Imperial Healthcare NHS Trust, Du Cane Road, London, W12 0HS UK; 3 Department of Development and Regeneration, KU Leuven, Leuven, Belgium; 4 Department of Biomedical and Clinical Sciences, University of Milan, Milan, Italy; 5 Department of Woman, Mother and Neonate, Buzzi Children Hospital, University of Milan, Milan, Italy

**Keywords:** Blood pressure, Pregnancy, Placenta, Vascular resistance, Beta blockers, Calcium channel inhibitors, Vasodilator therapy

## Abstract

Blood pressure is a way of describing the end result of changes in cardiac output, intravascular volume and peripheral resistance. It has certain advantages in that it is a reproducible and easily measured parameter, but in itself, it offers only a limited understanding of the underlying haemodynamics. In pregnancy, profound haemodynamic changes occur and in hypertensive diseases of pregnancy defining a condition by blood pressure alone risks missing the underlying cause. Partly, this has been a problem of ascribing the cause of hypertensive syndromes to the placenta which has inhibited rigorous research into other possible causes of haemodynamic dysfunction. It is becoming increasingly evident that hypertension in pregnancy may be associated with primarily high cardiac output or high peripheral resistance. A knowledge of the underlying type of hypertension may allow more rational treatment of these conditions in pregnancy rather than therapeutic attempts at controlling blood pressure by any means possible as an end in itself.

## Introduction

### Hypertension in Pregnancy: Definitions and History

Hypertension in pregnancy remains a ‘cinderella area’ of pathophysiology in clinical obstetrics. For many decades—and still now—the acute pathological changes that compromise the maternal cardiovascular system during pregnancy with possible devastating tissue and organ dysfunction remained firmly rooted to the nineteenth century criteria of high blood pressure and proteinuria, both ‘tertiary, downstream features of the disease’ [1]. The profusion of names for this condition, pre-eclampsia, toxaemia, gestosis and proteinuric pregnancy induced hypertension, underline the task of understanding such a condition whose diagnosis is based purely on the easily measurable manifestations of complex, possibly distinctly different causes of high blood pressure in pregnancy.

Let us consider the very name pre-eclampsia, literally a disease that occurs prior to eclampsia (‘προ-ἐκλαμψία’, ‘before-sudden occurrence’). This term was introduced by Johannes Varandaeus in the year 1620 treatise ‘Tractatus (of gynaecology) de affectibus Renum et Vesicae’. More than one century later, proteinuria was described in relation to fluid accumulation during pregnancy resulting in acute fits and possibly death if the fetus and placenta are not delivered. Pierre Rayer (1793–1867), a Frenchman, was the first to describe proteinuria in eclamptics, cited in his then classic text ‘Diseases of the Kidney’ [2]. It took a few more years for Scipione Riva-Rocci’s mercury manometer (1896) to be introduced and allowed to recognize that pre-eclampsia was a hypertensive disorder. It is surprising that in 2017, this condition is still classified according to Varandeus-Rayer and Riva Rocci. It is tempting to speculate that the consequences of defining hypertensive disorder according to such arcane criteria (ICD-10 code 014) have prevented a rational categorization of the condition based on cause, not effect.

But names and definitions are important. Indeed, some guidelines include almost everything from headache to liver damage to disseminated intravascular coagulation; intra uterine growth restriction (IUGR) plus hypertension qualifies for pre-eclampsia (PE) even without proteinuria. Broader names better fit broader criteria. We opt here for considering all these conditions within the collective term hypertensive disorders of pregnancy (HDP). The definition of HDP covers pregnancy induced hypertension, otherwise known as gestational hypertension (GH), all conditions of GH associated with maternal and foetal complications, among which can occur a typical renal glomerular lesion that causes proteinuria, and chronic hypertension (CH) (existing prior to pregnancy).

### The ‘Trophoblast Invasion’ Aetiological Theory

In 1975, Brosens I and Renaer M [3] published a key paper on ‘The pathogenesis of placentae infarcts in preeclampsia’. These concepts were later developed by Khong TY, De Wolf F, Robertson WB and still among the authors, Brosens [4]. In this latter work, pathologic findings on pre-eclamptic placentae were associated with small-for-gestational-age infants. This particular link between shallow trophoblastic invasion in early gestation, placental insufficiency, led to a focused scientific insight into this possible origin of maternal endothelial dysfunction and hypertension associated with IUGR. The link between such characteristics of poor placental development in the first trimester and ‘preeclampsia’ occurring early in gestation associated with IUGR was reviewed by Burton, Woods, Jauniaux and Kingdom in 2009 [5]. Doppler interrogation of uterine arteries has for three decades been widely studied as a possible proxy of these pathological changes occurring in early placental development: foetal growth and maternal hypertension [6–8].

Unfortunately, the theory [8], molecular [9] and histopathological [5] had hardly developed scientifically in 30 years and has persuaded many to consider this clinical phenotype the sole cause of pre-eclampsia by those that uncritically accepted association as equivalent to causation. Time at onset of the clinical signs and symptoms (before and after 34 weeks of gestation) became the criterion on which to define its severity. ‘Consequently, PE could be considered as a single pathophysiological entity with a wide spectrum of severity manifested in gestational age at which delivery becomes necessary for maternal and/or foetal indication’ [10]. This interpretation of pre-eclampsia is in striking contrast with epidemiological, pathological and clinical evidence and has arguably held up the proper understanding of the origin of both hypertensive disease and fetal growth restriction which are frequently closely related.

### Pathophysiology and Clinical Phenotype: Towards a New Definition

Apparently anomalous characteristics of the condition are that whilst in early onset pre-eclampsia, birthweight is reduced, at term the condition is associated with normal or large weight babies [11–13, 14•]. Other possible explanations—for example underlying cardiovascular dysfunction or metabolic syndrome—do not contradict the relationship between early placental shallow trophoblast invasion and subsequent maternal endothelial damage. They do however suggest a different clinical phenotype of pre-eclampsia where maternal risk factors appears to play a predominant role, and both placental and newborn weight appears to be normal or even large. The existence of two main placental diseases had been recently reviewed by Redman and Staff [15]. Both placental lesions, early shallow trophoblastic invasion and late villi crowding generate a similar oxidative stress that damage the maternal endothelium that magnify underlying maternal risks; these might be so severe as to require delivery prior to 34 weeks of gestation [16].

The obvious limitation of current definitions is that irrespective of the type of hypertension, women with identical systolic pressures are considered the same as each other, when their haemodynamics (cardiac output, stroke volume, heart rate, vascular resistance and arterial function) may be significantly different. Importantly, all recent studies [17–21] on maternal haemodynamics suggest profound differences from normal pregnancies in cardiac output, vascular resistance and arterial function depending on the type of pregnancy hypertension and the gestation at which it occurs. The fundamental problem is that hypertension itself is characterized as the problem rather than the manifestation of an underlying cardiovascular dysfunction.

In the clinical arena, the ongoing debate on how to re-classify and interpret pre-eclampsia might be summarized by the recently published Co-Lab statement: ‘Despite intensive study, we have not been able to improve the management or early recognition of preeclampsia…. It is possible that within the syndrome, there may be different phenotypes with pathogenic pathways that differ between the subtypes. The capacity to recognize and to exploit different subtypes is of obvious importance for prediction, prevention, and treatment’ [22]. These strong statements that challenge the validity of the long trial of knowledge accumulated on pre-eclampsia were anticipated in 2010 by Steegers and co-workers on a review paper on the Lancet [23]: ‘Poor early placentation is especially associated with early onset disease (of pre-eclampsia before 34 weeks of gestation). Predisposing cardiovascular or metabolic risks for endothelial dysfunction,…, might dominate in the origins of late onset pre-eclampsia (after 34 weeks of gestation).’

### Cardiovascular Function and Pre-eclampsia

Cardiac output (CO) increases during pregnancy and plateaus in the late second and early third trimester, 1.5 L/min (31%) above non-pregnant values [24•]. At 28 weeks of gestation, the percentage of total maternal CO to the uterus is approximately 12% [25•]. This increase is sustained both by the increasing heart rate (HR) and stroke volume (SV). Multiparity and multifetal pregnancies significantly influence these indices. Diastolic blood pressure shows a decrease that already starts in early first trimester and extends until the late second trimester [24•]. Total vascular resistance (TVR) decreases until the early second trimester. As a consequence, we usually observe a decrease in diastolic blood pressure. Mean arterial pressure (MAP) starts to increase towards term as compared to non-pregnant data. In this adaptation process, the venous system increases in venous capacitance, due to enhanced venous distensibility, and mostly the splanchnic system accommodates the normal plasma volume expansion [26•]. A recent meta-analysis calculated an average increase in plasma volume of 45.6% [27•].

Cardiovascular remodelling involves the heart itself. The left ventricular mass (LVM) increases to values 30–40% above non-pregnant values with a concentric hypertrophy since the first two trimesters of pregnancy [28•]. This dramatic increase outperforms that achieved only by exercise by athletes, who might gain 25% eccentric remodelling [28•]. The left atrial diameter is already significantly enlarged during the first weeks of pregnancy [29•, 30•].

It is plausible that a case of pre-eclampsia that occurs earlier in gestation and is associated with fetal growth restriction is related to low cardiac output and high peripheral vascular resistance [31] with a much similar profile as observed in women with fetal growth restriction without HDP [32]. In cases of later and term gestation pre-eclampsia, babies tend to be larger and there is a predominantly high cardiac output, low peripheral vascular resistance and raised intravascular volume state [33]. Certainly, the clinical phenotype of a very ‘dry’, intravascularly depleted woman at 26 weeks with a growth restricted baby and conversely of a well-perfused oedematous woman with a bounding pulse and large baby at 38 weeks rings true: both have hypertension, but the mechanisms may be diametrically opposite.

### Implications for Pharmacotherapy

It is plausible to consider that if these diametrically opposed cardiovascular states both cause hypertension, then the pharmacological treatment for one condition will not necessarily cure the other. Beta blockers, combined alpha and beta blockers, calcium antagonists, adrenergic false transmitters, presynaptic alpha receptor modulators, nitrates and diuretics have all been used to treat hypertensive pregnancy. International guidelines (NICE, ACOG) have in recent years supported a simplification of the therapeutic options which in the UK favour labetalol as a first line agent, with alpha methyl dopa as second line, a place for calcium antagonists and almost no place for diuretics. An inherent difficulty is that though these agents have quite different modes of action, they are used interchangeably. Further, national guidance is based on little data: studies of hypertensive agents of the scale performed in the general adult population do not exist hence empirical advice is given on the basis of what seems to be safe clinical practice and unlikely to harm the foetus. The major problem is that hypertension per se is considered to be the goal of therapy, irrespective of the underlying cause. The recent CHIPS study [34] investigated whether ‘tight’ blood pressure control conferred a benefit in outcome in women with hypertension in pregnancy, concluding that it did not. Would it not have been instructive in determining benefit to understand the underlying differences in womens’ cardiovascular status and in this light the response to treatment?

Contrast this approach with rapidly evolving specialist adult hypertensive practice. Knowledge of whether the condition is ‘high output’ or ‘high resistance’ allows selection of the anti-hypertensive agent that will work in the most physiological way—not by targeting blood pressure—but more the underlying condition [35, 36]. Non-invasive devices, now readily available and validated in pregnancy, give accurate cardiac output and other data and allow profiling of arterial function in hypertension [37]. In a low cardiac output, high vascular resistance scenario, a negatively inotropic alpha/beta blocker such as labetalol may make the haemodynamics worse whilst achieving the therapeutic goal of lowering blood pressure; a calcium channel blocker may be far better. In the well-perfused term, woman with hypertension and a high cardiac output, labetalol and a diuretic may be therapeutically better suited than a vasodilator—where there is already vasodilatation. It may also be that similar to anti-hypertensive therapy in the general population, prescription of the ‘wrong’ drug for the haemodynamic abnormality present may, due to physiological responses to treatment, make women more likely to be resistant to subsequent therapies. This is not to mention the fetal-placental unit, where a reduction in perfusion might have serious consequences.

## Conclusions

The current therapeutic approach, a ‘one size fits all’, is certainly too simplistic: why not tailor therapeutic approaches to haemodynamic profile rather than blood pressure alone? It is now timely to define hypertensive conditions in pregnancy based on the cardiovascular profile, treat women and perform studies based on rational assessment of pharmacological action—not purely of anti-hypertensive efficacy.
